# Adaptive radiotherapy for head and neck cancer

**DOI:** 10.1186/s41199-019-0046-z

**Published:** 2020-01-09

**Authors:** Howard E. Morgan, David J. Sher

**Affiliations:** Department of Radiation Oncology, University of Texas Southwestern Medical Center, 2280 Inwood Rd, Dallas, TX 75390 USA

**Keywords:** Adaptive radiotherapy, Head and neck squamous cell carcinoma, IMRT, PET-guided radiotherapy, MRI-guided radiotherapy

## Abstract

**Background:**

Although there have been dramatic improvements in radiotherapy for head and neck squamous cell carcinoma (HNSCC), including robust intensity modulation and daily image guidance, these advances are not able to account for inherent structural and spatial changes that may occur during treatment. Many sources have reported volume reductions in the primary target, nodal volumes, and parotid glands over treatment, which may result in unintended dosimetric changes affecting the side effect profile and even efficacy of the treatment. Adaptive radiotherapy (ART) is an exciting treatment paradigm that has been developed to directly adjust for these changes.

**Main body:**

Adaptive radiotherapy may be divided into two categories: anatomy-adapted (A-ART) and response-adapted ART (R-ART). Anatomy-adapted ART is the process of re-planning patients based on structural and spatial changes occurring over treatment, with the intent of reducing overdosage of sensitive structures such as the parotids, improving dose homogeneity, and preserving coverage of the target. In contrast, response-adapted ART is the process of re-planning patients based on response to treatment, such that the target and/or dose changes as a function of interim imaging during treatment, with the intent of dose escalating persistent disease and/or de-escalating surrounding normal tissue. The impact of R-ART on local control and toxicity outcomes is actively being investigated in several currently accruing trials.

**Conclusions:**

Anatomy-adapted ART is a promising modality to improve rates of xerostomia and coverage in individuals who experience significant volumetric changes during radiation, while R-ART is currently being studied to assess its utility in either dose escalation of radioresistant disease, or de-intensification of surrounding normal tissue following treatment response. In this paper, we will review the existing literature and recent advances regarding A-ART and R-ART.

## Background

Over the past 20 years, the standard-of-care for radiotherapy of head and neck squamous cell carcinoma (HNSCC) has transitioned from 2D radiotherapy (RT) to 3D-conformal radiotherapy (3D-CRT) to intensity-modulated radiation therapy (IMRT) [1]. While IMRT has been shown to reduce normal tissue toxicities such as xerostomia [2], its dramatically improved conformality over 2D and 3D-CRT means that anatomic changes in the patient due to weight loss or tumor reduction may have a dramatic impact on the delivered dose. Indeed, many patients will experience volumetric and spatial changes of the target volumes and organs-at-risk (OAR) during treatment, which may be due to some combination of treatment response, weight loss, inflammation, muscle atrophy, and radiation effects on normal tissues. These changes are of significant importance to the dose actually received by the patient, as they are not accounted for on the initial planning scan.

For example, as patients progress through treatment, radiosensitive structures such as the parotids may migrate closer to high dose regions [3, 4] resulting in an unintended overdosage which has been associated with worse predicted xerostomia in small retrospective series [3], and target structures may develop dose inhomogeneities with unintended cold spots which have been associated with worse local control in non-randomized cohorts [5, 6]. Sophisticated image guidance technologies such as daily cone-beam computed tomography (CBCT) [7] can account for setup errors between treatment days, but they cannot adjust for inherent changes in the volume and spatial location of the tumor and normal tissues.

In response to this fundamental problem, adaptive radiotherapy (ART) has been developed to address these shortcomings. Adaptive radiotherapy is the process of re-planning patients during treatment either in response to a stimulus, such as weight loss or tumor shrinkage, or at pre-defined intervals over the course of radiation. The process of re-planning allows the radiation plan to adjust to the changing tumor and normal tissue anatomy, reducing dose to sensitive structures such as the parotid glands, while minimizing dose inhomogeneity and inadequate target coverage. In this scenario, ART can be referred to as anatomy-adapted adaptive radiotherapy (A-ART), given ART is guided by structural changes occurring over the course of radiation. In contrast, there has been recent interest in utilizing diagnostic imaging during treatment, such as PET/CT or MRI, to identify treatment response in the primary tumor and/or nodal volumes to guide dose escalation and de-escalation attempts. In this setting, ART can be referred to as response-adapted adaptive radiotherapy (R-ART), since ART is guided by response to therapy. The purpose of this article is to review the existing literature on anatomy- and response-adapted ART.

## Main text

### A-ART: impact of volumetric and spatial changes during radiotherapy on delivered dose

Several structures have been shown to change size and shape over the course of radiation for HNSCC, most notably the primary tumor, involved nodes, and the parotid glands. Many retrospective and prospective series have consistently reported decreased tumor size which can be detected as early as the first 2 weeks, with median reported shrinkage rates ranging from 3 to 16%, 7 to 48% and 6 to 66% reduction by the end of week 2 [8, 9], 4 [10–16], and 7 [3, 9, 15, 17–28], respectively. Involved nodes can also shrink throughout treatment to similar degrees as the primary tumor [9, 13, 14, 16, 22–25, 28]. Of note, tumor shrinkage during treatment is very heterogenous even within studies, which is not surprising given the known spectrum of radioresponsiveness in HNSCC. For example, one study reported a range of 79.6% reduction to − 18.8% increase in primary tumor volume by end of treatment among 34 patients receiving definitive chemoradiotherapy for HNSCC at the Loyola University Medical Center [16]. Another study reported a range of 73% reduction to − 13% increase in the high dose clinical target volume (CTV) by the end of treatment in 15 patients receiving definitive chemoradiotherapy for HNSCC [3] in France. For any given patient with a significant volumetric change, though, there may be significant consequences in delivered dose inhomogeneity [11, 15], potentially resulting in overdosage of normal tissues or underdosage of the target structures [18, 29]. See Table 1 for a summary of the volumetric changes of the primary tumor volume in HNSCC over the course of radiotherapy.

**Table 1:** Primary tumor volumetric changes in HNSCC or NPC during curative RT or CRT

Study	Sample Size (n)	Re-Scan Timing	Mean Primary Tumor Volume Reduction (Range) by Fraction (Fx) 10	Mean Primary Tumor Volume Reduction (Range) by Fx 20	Mean Primary Tumor Volume Reduction (Range) by End of Treatment
Bhide IJROBP 2010 [8]	20 (10 OPC, 6 Laryng or Hypoph, 4 NPC)	Weeks 2, 3, 4, 5	3.2% CTV1, where CTV1 was gross disease + 1.5 cm margin (0.74–5.6%)	–	–
Liu IJROBP 2018 [9]	18 (16 OPC, 1 NPC, 1 UP)	Fx 10 and 22	16.4% GTV, 15.4% CTV1 where CTV1 was GTV + 0.5 cm margin (GTV − 2.1 to 37.1%, CTV1 0.7 to 30.5%)	–	32.6% GTV, 28.2% CTV1 by fraction 22 (GTV 9.8 to 56.3%, CTV1 12.0 to 44.8%)
Capelle Clin Oncol 2012 [10]	20 (17 HNSCC, 3 NPC)	Fx 15	–	28.8% GTV, 16% PTV60/66 where PTV60 was a 0.5 cm expansion on CTV60 (involved postop regions) for adjuvant patients and PTV66 was a 0.5 cm expansion on the GTV for definitive patients (GTV 1.6 to 60%, PTV 0 to 45%)	–
Chitapanarux J Radiat Res 2015 [11]	17 (17 NPC)	Fx 17	–	18.4% GTV, − 7.4% CTV70 by fraction 17 where CTV70 was GTV + 0.5 cm margin (range NS)	–
Dewan Asian Pac J 2016 [12]	30 (15 OPC, 10 OC, 5 Hypoph)	Fx 20	–	47.62% GTV, 43.76% CTV where CTV was GTV + 0.5–2 cm margin, 49.69% PTV where PTV was 0.2–0.5 cm margin by fraction 20 (range NS)	–
Lee Cancer Res Treat 2016 (13)	159 (159 NPC)	Fx 15	–	43.4% GTV (− 3.8 to 93.5%)	–
Lu Chin Med J 2012 [14]	43 (43 NPC)	Fx 20	–	30.1% GTV by Fraction 20 (0.6 to 77.2%)	–
Mahmoud Technol Cancer Res Treat 2017 [15]	22 (11 OPC, 7 OC, 2 Laryng, 1 Hypoph, 1 NPC)	Fx 15, 27	–	7.2% CTV-HR for definitive (− 3 to 18%) 7.8% CTV-HR for postoperative (0 to 18%) where CTV-HR “included the regions and/or subjacent lymph node chains within 2 to 3 cm of gross disease”	12.8% CTV-HR for definitive (−7 to 29%) 10.9% CTV-HR for postoperative (3 to 20%)
Surucu Technol Cancer Res Treat 2016 [16]	48 (28 OPC, 7 NPC, 5 Hypoph, 8 OC)	Week 4 (median; rescans done at a median dose of 37.8Gy with range of 14.4–51.5 Gy)	–	26.8% GTV (range NS)	–
Surucu Technol Cancer Res Treat 2017 [27]	34 (20 OPC, 6 OC, 5 NPC, 3 Hypoph)	Week 4 (median; rescans done at a median dose of 37.8Gy with range of 27.0–48.6 Gy)	–	–	35.2% GTV (−18.8 to 79.6%)
Castelli Radiat Oncol 2015 [3]	15 (11 OPC, 2 OC, 1 Laryng, 1 Hypoph)	Weekly	–	–	31% CTV70 where CTV70 was GTV + 0.5 cm margin (−13 to 73%)
Loo Clin Oncol 2011 [17]	5 (3 OPC, 1 OC, 1 Laryng)	Weekly	–	–	5.8% CTV68 where CTV68 “encompassed the GTV and high-risk regions” (range NS)
Beltran J Appl Clin Med 2012 [18]	16 (7 OPC, 5 OC, 1 Hypoph, 1 NPC, 2 NS)	Fx 15, 25	–	–	13.25% PTV2 where PTV2 was the CTV2 (GTV + high-risk regions) + 0.5 cm margin (range NS)
Fung Med Dosim 2012 [19]	10 (10 NPC)	Fx 21, 31 approx.	–	–	53.95% CTV, 36.19% PTV where PTV was the CTV + 0.3 cm (range NS)
Jin Radiat Oncol 2013 [20]	9 (9 NPC)	Fx 23	–	–	9.4% GTV by fraction 23 (range NS) (non-sig reduction)
Schwartz Radiother Oncol 2013 [21]	22 (22 OPC)	Daily scans, Weekly recalc	–	5% CTV where CTV was the GTV + high-risk regions (−21 to 13%)	8% CTV (−6 to 19%)
Tan Onco Targets Ther 2013 [22]	20 (20 NPC)	Weekly	–	–	55.3% GTV (range NS)
Fung J Radiat Res 2014 [23]	30 (30 NPC)	Every 2 Fx	–	–	35.7% GTV (range NS)
Huang Radiat Oncol 2015 [24]	19 (19 NPC)	Every 5 Fx	–	–	65.6% GTV (range NS)
Kataria Br J Radiol [25]	36 (21 OPC, 5 Laryng, 10 Hypoph)	Fx 23	–	–	34.0% GTV (range NS)
Zhang Radiother Oncol 2016 [26]	13 (13 OPC)	Weekly	–	–	24.43% CTV70 where CTV70 was GTV + 0.5 cm margin (−12.6 to 62.1%)
Range of median tumor volume (GTV / CTV / PTV) reductions of the included studies			3 to 16%	7 to 48%	6 to 66%

Most studies reported a reduction in the primary target volume over the course of radiotherapy. However, the studies varied in the definition of the target volume reported, with some reporting the GTV, some the high risk CTV (with varying margins), and some the high risk PTV (also with varying margins), making comparisons across studies difficult. Note that for studies that included a wide range of fractions reported at time of re-scan, the median fraction was used for categorizing into the above columns (by Fx 10, Fx 20, etc). In regards to Bhide IJROBP 2010 (8) some patients had induction chemotherapy prior to definitive CRT, which may account for some of the variation seen between the volumetric changes reported by this author and by Liu IJROBP 2018 (9). Overall, the trend seen in these series was for increasing tumor volume reduction throughout therapy with median reductions reported as 3 to 16% by fraction 10, 7 to 48% by fraction 20, and 6 to 66% by end of treatment

“- “information was either not available or was not directly comparable to other volumetric/dosimetric data reported and thus not included

*GTV* Gross Tumor Volume, *CTV* Clinical Target Volume, *PTV* Planned Target Volume, *HNSCC* Head and Neck Squamous Cell Carcinoma, *OPC* Oropharyngeal Cancer, *OC* Oral Cavity Cancer, *NPC* Nasopharyngeal Cancer, *Laryng* Laryngeal Cancer, *Hypoph* Hyopharyngeal Cancer, *NS* Head and Neck Squamous Cell Carcinoma, Site Not Specified, *SN* Sinonasal Cancer, *UP* Head and Neck Squamous Cell Carcinoma of Unknown Primary, *Range NS* Range not stated

In fact, there has been significant interest in the need for ART to improve local control in the definitive treatment of HNSCC [18, 29]. If ART could correct for these inhomogeneities, then cold spots would be minimized and hypothetically improve in-field failure rates. No randomized data currently exists comparing oncologic outcomes of adaptive and non-adaptive plans to verify this assertation. Retrospective data does appear to suggest a benefit. In a study of 317 patients receiving definitive or adjuvant radiation for HNSCC at UC Davis [5], 51 patients who underwent A-ART per clinical discretion were compared with those who were not re-planned, and there was a significantly higher rate of 2 year local-regional control with A-ART (88% vs 79%, *p* = 0.01). Of note, all of the local failures within the A-ART group were in-field of the primary PTV. In a separate propensity score matched analysis, 66 patients receiving definitive CRT for T3/T4 NPC with A-ART were matched with 66 patients without A-ART and found that 5 year local-regional recurrence-free survival was higher in those receiving A-ART (96.7% vs 88.1%, *p* = 0.022) [6], but with the major pattern of failure being distant metastases which did not differ significantly between groups. Both of these studies are limited by a lack of standardization of adaptive re-planning specifications and their non-randomized study design. For example, if tumor response was used as a cue to initiate ART, then the use of it would likely select for patients more likely to achieve a partial or complete response following completion of treatment [13].

With respect to OARs, the parotid glands are of particular importance in A-ART, as their radiosensitivity is clearly established, resulting in decreased salivary output at low doses of radiation with associated xerostomia and reduced quality-of-life [30]. In 1999, Eisbruch and colleagues [31] demonstrated that mean doses to the parotid glands as low as 26 Gy can lead to irreversible xerostomia. With the advent of IMRT, treatment plans were able to spare the parotid glands while still conforming to the target and obtaining adequate coverage. Both contralateral parotid sparing as assessed in PARSPORT I [2] and bilateral superficial parotid sparing methods as assessed in PARSPORT II [32, 33] have shown promising results in regards to minimizing xerostomia following definitive RT for HNSCC. However, not all patients who appear to have excellent sparing of the parotids on treatment planning have excellent rates of xerostomia, as 38% receiving IMRT in PARSPORT I [2] and 21% in PARSPORT II still had grade 2 or greater xerostomia by month 12 [32]. Whether this residual xerostomia is fully due to inherent differences in patient response to RT is unclear, but unrecognized (and therefore unadjusted) changes in parotid dosimetry throughout treatment may partially contribute.

**Fig. 1:**
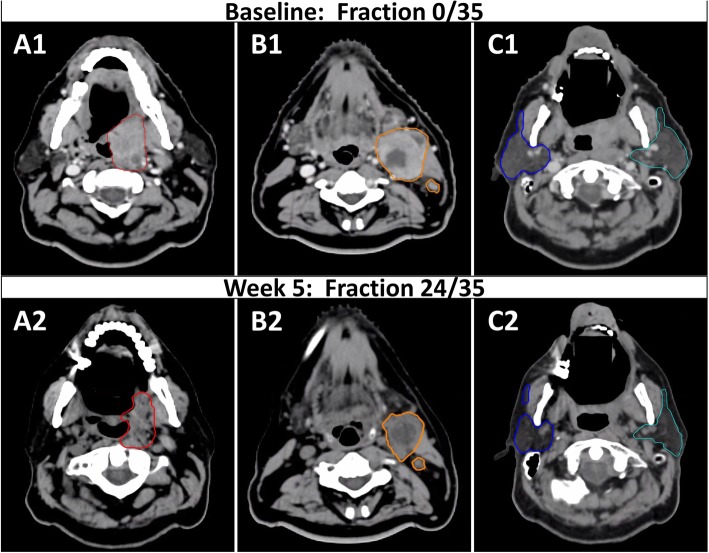
Primary tumor, nodal, and parotid volumes decrease over the course of radiation. This patient is a 54 year-old man with p16-positive cT4N1M0 squamous cell carcinoma of the left tonsil who required adaptive radiotherapy over the course of radiation secondary to significant tumor response and weight loss during treatment noted on review of daily CBCTs. The primary tumor decreased by 25.0% from baseline (A1) to week 5 (A2). The grossly involved nodes decreased by 48.6% from baseline (B1) to week 5 (B2). The left parotid decreased by 37.2% (cyan) and the right parotid (blue) decreased by 41.9% from baseline (C1) to week 5 (C2). Note contraction of the lateral border of the bilateral parotids at time of re-simulation (C2)

Like the primary tumor and involved nodes, the parotid glands have also been consistently reported to shrink throughout treatment, a process that may start as early as the first 2 weeks of treatment. The average volume of the parotids has been reported to decrease as much as 14.7, 37, and 48% by the end of weeks 2 [8], 4 [10–12, 14, 15, 18, 21, 24, 34], and 7 [3, 8, 11, 15, 17–21, 23, 24, 34–37], meaning that the delivered dose could be much higher than expected by the original plan. Like the target volumes, there can also be wide heterogeneity in the volume reduction of parotid glands. One study reported a range of 0.0 to 63.4% reduction by end of treatment [3] while another reported a range of 6.8 to 69.44% reduction by end of treatment [36]. This heterogeneity between patients likely contributes to the seemingly contradictory findings between some small studies which predict a xerostomia reduction benefit of A-ART [3, 38] and some studies which do not [35, 39]. See Table 2 for a summary of volumetric and dosimetric changes of the parotids in HNSCC. Figure 1 is an example of a patient who might benefit from A-ART.

**Table 2:** Parotid volumetric and dosimetric changes in HNSCC or NPC during curative RT or CRT

Study	Sample Size (n)	Re-Scan Timing	Mean Parotid Volume Reduction (Range) by Fraction (Fx) 10	Mean Parotid Volume Reduction (Range) by Fx 20	Mean Parotid Volume Reduction (Range) by End of Treatment	Dosimetric: Mean Parotid Dose Change (Range)
Bhide IJROBP 2010 [8]	20 (10 OPC, 6 Laryng or Hypoph, 4 NPC)	Weeks 2, 3, 4, 5	14.7% ^NS^	–	35% ^NS^	Without ART: 2.8 Gy increase ^ipsi^
Capelle Clin Oncol 2012 [10]	20 (17 HNSCC, 3 NPC)	Fx 15	–	17.5% ^NS^ (−1 to 46%)	–	With ART: 0.6 Gy reduction ^NS^
Lu Chin Med J 2012 [14]	43 (43 NPC)	Fx 20	–	35.5 to 36.8% *	–	–
Beltran J Appl Clin Med 2012 [18]	16 (7 OPC, 5 OC, 1 Hypoph, 1 NPC, 2 NS)	Fx 15, 25	–	22% ^NS^	30% ^NS^	Without ART: 4.7% increase ^Ipsi^ 6.1% increase ^Contra^
Schwartz Radiother Oncol 2013 [21]	22 (22 OPC)	Daily scans, Weekly recalc	–	15% ^NS^ (−19 to 25%)	26% ^NS^ (−8 to 48%)	1 ART re-plan: 1.3 Gy reduction ^ipsi^ 2 ART re-plans: 4.1 Gy reduction ^ipsi^
Chitapanarux J Radiat Res 2015 [11]	17 (17 NPC)	Fx 17	–	30.5% ^ipsi^ 24.3% ^contra^	–	With ART: 1.1 Gy reduction ^Contra^ Ipsilateral not significant (0.9 Gy)
Huang Radiat Oncol 2015 [24]	19 (19 NPC)	Every 5 Fx	–	14.4 to 15.8% *	38.0 to 39.2% *	Without ART: 3.09 Gy to 5.6 Gy increase *
Dewan Asian Pac J 2016 [12]	30 (15 OPC, 10 OC, 5 Hypoph)	Fx 20	–	33.65% ^ipsi^ 31.06% ^contra^	–	With ART: 5.6 Gy reduction ^ipsi^ Contralateral not significant (2.7 Gy)
Zhang J Med Radiat Sci 2017 [34]	39 (39 NPC)	Fx 10, 20, 30	–	15.27% ^ipsi^	37.49% ^ipsi^ 34.55% ^contra^	–
Mahmoud Technol Cancer Res Treat 2017 [15]	22 (11 OPC, 7 OC, 2 Laryng, 1 Hypoph, 1 NPC)	Fx 15, 27	–	18.2 to 19.0% * for definitive (3 to 32%) 10.0 to 16.6% * for adjuvant (5 to 44%)	30.1 to 30.9% * for definitive (11 to 52%) 23.1 to 25.3% * for adjuvant (3 to 41%)	Without ART: 15.4 to 16.4% increase * for definitive (−30 to 76%) 9.1 to 10.4% increase * for postop (−25 to 70%) Neither significant given large heterogeneity
Loo Clin Oncol 2011 [17]	5 (3 OPC, 1 OC, 1 Laryng)	Weekly	–	–	30.2% ^Ipsi^ (17.1 to 55.8%) 17.5% ^Contra^ (15.6 to 48.5%)	Without ART: 7.6 Gy increase ^Ipsi^ (2.5 to 19 Gy) 7.3 Gy increase ^Contra^ (1.1 to 11.6 Gy)
Fung Med Dosim 2012 [19]	10 (10 NPC)	Fx 21, 31 approx.	–	–	32.44 to 33.31% *	With ART: 0.75 Gy reduction (right, *p* = 0.046) 1.25 Gy reduction (left, *p* = 0.053)
Fung J Radiat Res 2014 [23]	30 (30 NPC)	Every 2 Fx	–	–	47.54% ^NS^	–
Hunter IJROBP 2013 [35]	18 (18 OPC)	Weekly	–	–	13.31% ^NS^	Without ART: 0.92 Gy (median) increase ^NS^ (−4.9 to 8.4 Gy; not sig) 23/36 parotids had an increase (2.2 Gy median)
Jin Radiat Oncol 2013 [5]	9 (9 NPC)	Fx 23	–	–	38.4 to 40.68% *	–
Castelli Radiat Oncol 2015 [3]	15 (11 OPC, 2 OC, 1 Laryng, 1 Hypoph)	Weekly	–	–	28.3% ^NS^	Without ART: 67% of pts.: 4.8 Gy increase ^NS^ 33% of pts.: 3.9 Gy decrease ^NS^ With ART: Of those with overdosed parotids: 5.1Gy decrease ^NS^ (0.6 to 12.2 Gy)
Yao Biomed Res Int 2015 [36]	50 (50 NPC)	Every 5 Fx	–	–	35% ^NS^ (6.8 to 69.4%)	Without ART: 3.52 Gy (11.38%) increase ^NS^ (−1.51 to 30.57%) Weight loss correlated with mean parotid dose
Zhang Radiother Oncol 2016 [26]	13 (13 OPC)	Weekly	–	–	34.51% ^ipsi^ (10 to 57.6%) 27.98% ^contra^ (−5.2 to 57.3%)	Without ART: 16/23 parotids overdosed: 4.1 Gy increase ^NS^ (0.5 to 11.5 Gy) 3 ART re-plans: 3.1 Gy reduction ^NS^ 6 ART re-plans: 3.3 Gy reduction ^NS^
Hu BMC Cancer 2018 [40]	40 (40 NPC)	Median Fx 22	–	–	17.2% ^Ipsi^ 20% ^Contra^	With ART: 0.7 Gy reduction ^Ipsi^ Contralateral not significant (0.1 Gy)
Lee IJROBP 2008 [4]	10 (2 OPC, 5 NPC, 1 SN, 1 OPC + NPC, 1 UP)	Daily MV-CT	–	–	–	Without ART: 3 Gy (11%) NS (−6 to 42%) Parotid glands migrating closer to target volume had higher changes in mean dose
Fiorentino Br J Radiol 2012 [41]	10 (4 OPC, 5 OC, 1 Hypoph)	Daily	–	–	43.5% ^Ipsi^ 44.0% ^Contra^	–
Range of median parotid volume reductions reported in the included studies			15%	10 to 37%	13 to 48%	–

All studies reported an average decrease in parotid volume at time of re-scan; however, there was wide heterogeneity between and even within studies, with a few patients actually having an increase in parotid gland volume by end of treatment. This was associated with variable reductions in mean parotid dose by adaptive re-planning and suggests that ART may not be appropriate for all patients. However, ART does appear to reduce mean parotid dose in patients whose parotids experience an unintended overdosage secondary to anatomic changes throughout treatment, which has been associated with reduced predicted xerostomia. However, clinical correlation is still lacking between ART and prospective toxicity data. Note that a negative volumetric change reported above means that this structure increased in size (e.g. -1% indicates a 1% increase in volume). A negative dosimetric change means that it decreased in the dose received (e.g. -10% indicates a 10% decrease in mean parotid dose)

“- “information was either not available or was not directly comparable to other volumetric/dosimetric data reported and thus not included

“^NS^” parotid side was not specified

“* “parotid side (left or right) was specified; however, ipsilateral and contralateral designation were not specified

“^Ipsi^ “ipsilateral parotid

“^Contra^ “contralateral parotid

*HNSCC* Head and Neck Squamous Cell Carcinoma, *OPC* Oropharyngeal Cancer, *OC* Oral Cavity Cancer, *NPC* Nasopharyngeal Cancer, *Laryng* Laryngeal Cancer, *Hypoph* Hyopharyngeal Cancer, *NS* Head and Neck Squamous Cell Carcinoma, Site Not Specified, *SN* Sinonasal Cancer, *UP* Head and Neck Squamous Cell Carcinoma of Unknown Primary

**Fig. 2:**
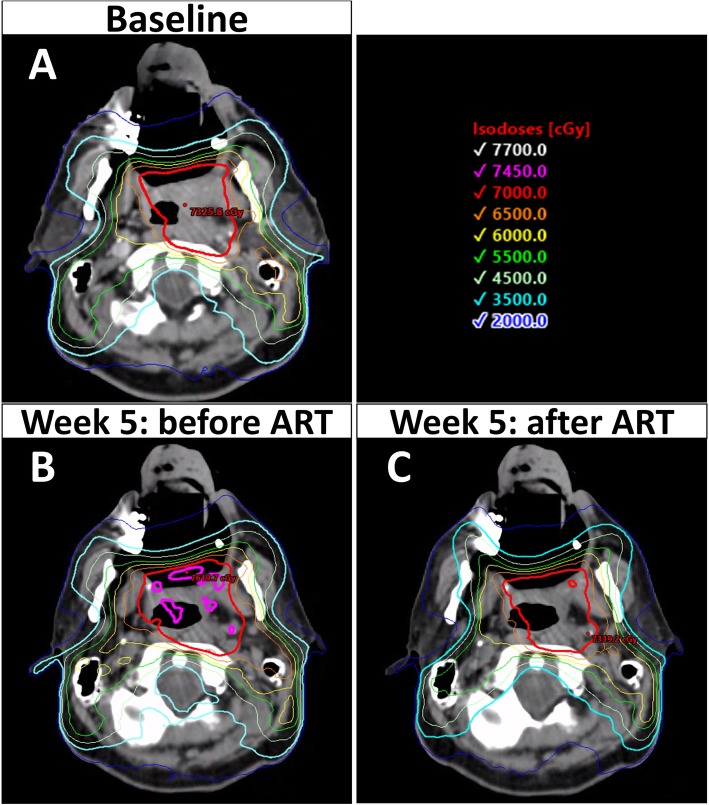
Adaptive re-planning reduces unplanned dose inhomogeneity and parotid gland overdose. These images are from the same case as presented in Fig. 1. At time of initial simulation (**a**), anticipated coverage of the high dose planned target volume (PTV) was 98.5% receiving 70Gy and the mean dose of the left and right superficial parotids were 25.0 and 24.5 Gy, respectively. However, by week 5 (**b**), there was wide variation in dose within the high dose PTV with cold spots down to 88.0% and hot spots up to 113.4% of the prescription. In addition, the mean left and right superficial parotids doses increased to 32.2 Gy and 36.7 Gy, respectively. With adaptive re-planning (**c**), dose homogeneity was improved with cold spots only being 94.8% and hot spots only being 104.4% inside of the high dose PTV, with reduction of the mean right and left superficial parotid dose back to 24.9 Gy and 24.6 Gy, respectively. The main benefit of A-ART in this case was sparing of the parotids, given there was an unplanned overdose of an additional 7.2 Gy to the left and 12.2 Gy to the right parotids which was mitigated with adaptive re-planning

In addition to volume changes, the spatial orientation of the parotid glands appears to shift during treatment, with a typical pattern of superior and medial displacement [3, 4, 8, 23, 24, 34] thought from shrinkage of the target tumor and associated weight loss. The implication of this shift means that the parotid gland may migrate closer to high-dose regions, resulting in an unplanned overdosage of this structure. In a retrospective cohort of 15 locally advanced HNSCC (primarily oropharyngeal) receiving definitive CRT, Castelli and colleagues [3] noted that 74% of the parotid glands were an average of 4.3 mm closer to the CTV by the end of treatment when compared to the initial planning scan. This was associated with an unplanned overdose of 59% of the parotid glands with an average mean dose increase of 3.7 Gy, with A-ART re-planning reducing the mean parotid dose by 5.1 Gy on average, with a predicted decrease in xerostomia risk of 11% based on a normal tissue complication probability (NTCP) model [3]. Other studies have also noted increased dose to the parotids with migration medially [24] towards the target [4]. Again, the degree of migration between patients is heterogenous with one study reporting parotid glands moving between 12 mm closer to the CTV to 6.2 mm away [3]. Given patients appear to differ fairly broadly in the degree of target shrinkage, parotid volume reduction, and parotid displacement, the subgroup of patients with minimal volumetric and spatial changes over the treatment course would likely gain little from proactive adaptive re-planning. Therefore, the identification of a cohort of patients who are most likely to benefit from A-ART is of significant interest to identify the appropriate population for re-planning, as this process is currently labor-intensive. Figure 2 shows an example of the relative dosimetric improvement of A-ART.

In addition to the parotid glands, the spinal cord and brainstem are also of interest as hot spots may develop in them over the course of radiotherapy, which may exceed conventional dosimetric constraints, that have been chosen to keep myelopathy and brainstem necrosis rates negligible. Most authors advocate for re-planning when these constraints (spinal cord max dose < 45–48 Gy and brainstem max dose < 54 Gy) are breached during radiotherapy. However, studies do not consistently report overdosing of the spinal cord or brainstem with some reporting significant increases in the max dose throughout radiotherapy [8, 10, 11, 19, 24, 42] and some noting no change [20, 40, 43, 44]. In a prospective cohort study of 22 patients with HNSCC where re-planning was triggered by underdosage of the target volumes (CTV coverage < 95%), overdosage of the parotids (mean dose > 26 Gy), or overdosage of the spinal cord (max dose > 45 Gy) [15], the spinal cord max dose reached the threshold for triggering A-ART in only 3 of 22 patients, whereas parotid gland overdosages occurred in 3 right and 5 left parotids, and CTV undercoverage in 7 instances. In contrast to the parotids, the volume and position of the spinal cord has not been shown to change over the course of radiotherapy [17, 45] which may partially explain why dosimetric changes in the spinal cord and/or brainstem are not as consistent or profound. However, in the studies that do report excess dose to the spinal cord and/or brainstem, dose variation can be quite high with one study reporting a range of 0.2–15.4 Gy increase in the spinal cord max dose and 0.6–8.1 Gy increase in the brainstem [42] and 2.1–9.9 Gy and 1.6–5.9 Gy respectively in another [11]. Note that a max dose increase of 15 Gy in the spinal cord can be quite significant, as rates of myelopathy increase exponentially at higher doses, with the estimated risk being < 1% at 54 Gy and < 10% at 61 Gy [46]. In individuals who encounter hot spots in the spinal cord or brainstem during radiotherapy, re-planning has consistently been reported to decrease these max doses back to within their pre-defined thresholds [11, 19, 42]. Though A-ART is beneficial in negating hot spots that may develop in the spinal cord and/or brainstem over the course of radiotherapy, the clinical significance of this is unclear as myelopathy rates remain very low in clinical practice. Further, most patients appear to only have modest increases in the spinal cord max dose (2–4 Gy) [11, 24, 42] and with several series failing to show a significant overdosage of this structure [20, 40, 43, 44]; clinically significant deviations may only occur in a minority of patients.

The effect of adaptive re-planning on the efficacy and tolerability of postoperative radiation for HNSCC is less clear, given the scarcity of data in this cohort. Some authors have advocated for A-ART in the postoperative setting noting that the postsurgical graft can swell and contract during radiation resulting in under-coverage [5]. However, other studies have questioned the utility of A-ART in the postoperative setting. In a prospective study of 20 patients with HNSCC who were re-planned at fraction 15, the 7 patients receiving postoperative radiation only appeared to have incremental, minimally impactful, changes in dosimetry [10]. Another more recent study prospectively assessed 22 patients with HNSCC but performed CTs for dose recalculation at weeks 1, 3, and 6 to determine the need for A-ART [15]. In this study, 2 of the 11 patients receiving postoperative radiation were re-planned as indicated by critical dosimetric changes by week 6, with one event triggering A-ART by the development of a spinal cord hot spot and one by CTV underdosage. In contrast, 8 of the 11 in the definitive group with bulky disease (gross tumor > 4 cm) encountered at least one event triggering A-ART which was significantly higher than the postoperative group (*p* = 0.03). Given that patients receiving definitive radiation had more weight loss (8.6% vs 4.9%, *p* < 0.001) and a trend towards more high risk CTV shrinkage (12.8% vs 10.9%, *p* > 0.05), the authors speculated that target shrinkage and weight loss may help explain the higher incidence of A-ART triggers in the definitive group, though comparisons are limited given the small sample size and lack of clinical correlation with A-ART trigger endpoints. Therefore in the setting of postoperative radiation, because the disease has been resected and the primary driver of anatomic change appears to be weight loss rather than tumor shrinkage, A-ART appears to be needed less frequently in patients treated with adjuvant RT.

Recently, MRI-guided radiotherapy has been evaluated for its utility in A-ART [47, 48], with the idea that adaptive scans utilizing MRI imaging can dramatically improve visualization of soft tissue changes throughout treatment. In a prospective feasibility study at MD Anderson Cancer Center [48], five patients with locally advanced HPV-positive OPC underwent definitive CRT, with intra-treatment MRI every 2 weeks. The primary gross tumor volume (GTV) volume was noted to decrease on average by 44, 90, and 100% at weeks 2, 4, and 6, with the corresponding nodal volumes decreasing by 25, 60, and 80%. The high dose target volume was reduced accordingly with these volumetric changes, resulting in an approximate reduction in the mean parotid dose of 3.3 Gy with ART. Although NTCP modeling only predicted a 1% xerostomia reduction at 6 months, the probability of predicted 6 month dysphagia was reduced by 11% [48]. The high rates of radiographic complete response (CR) on MRI of the primary tumors in this study are congruent with a prior pilot trial by the same group where 15 out of 29 primary tumors had a CR detected on MRI imaging at 3–4 weeks [47]. A separate group reported 70% average GTV reduction on MRI imaging by week 6 in eight patients with OPC [49]. The MARTHA trial is an upcoming prospective trial attempting to assess if the use of daily MRI imaging and weekly plan adaptation will benefit xerostomia in patients receiving RT for HNSCC [50].

### A-ART: practical considerations and implementation

Currently, the process of A-ART requires (1) identifying the appropriate patient, (2) re-simulation, (3) re-contouring, and (4) re-planning. Patients may be identified for A-ART by clinical variables (weight loss, tumor shrinkage, etc); regularly planned intervals; treatment response as assessed on CBCT scans, diagnostic CT or MRI scans; or dose re-calculations of cumulative dose delivered to the targets and OARs. Following identification, re-simulation of the patient should occur promptly, which may require creation of a new aquaplastic/thermoplastic mask if the mask fit is inadequate. Then, re-contouring can be done via manual input from the physician, deformable image registration, or automatic segmentation. Artificial intelligence methods of auto-contouring are being developed to make this process more efficient. If deformable image registration or an automatic method are used for re-contouring, it is recommended that the physician proofread these contours for errors prior to approval. The plan is then re-planned and optimized per physician discretion.

One of the biggest obstacles with adaptive re-planning is the time required to manually re-simulate, re-contour, and re-plan patients, which can be draining on a department’s resources; developing an optimal trigger for implementing A-ART is therefore a high priority to maximize efficiency. At this time, no consensus exists on the most appropriate timing regimen for A-ART during radiotherapy. Many centers perform adaptive re-planning based on clinical characteristics, such as weight loss, tumor shrinkage, changes in patient setup, and mask fitting. Other approaches suggest performing A-ART at regular intervals (e.g. every 10 fractions), as reductions in target and parotid volumes have been shown to occur as early as the first or second week which can result in significant dosimetric changes [24, 26]. In a study assessing the timing of A-ART scans in 13 patients with OPC receiving definitive radiation [26], weekly CT scans were performed and assessed for a dosimetric benefit of A-ART re-planning at each interval. They found 3 re-plans (weeks 1, 2, and 5) to be comparable with 6 weekly re-plans estimating a mean parotid gland benefit of 3.1 Gy with 3 re-plans compared with only 3.3 Gy if 6. The majority of the benefit appeared to be within the first 2 weeks, with the authors recommending A-ART at weeks 1, 2, and 5 [26]. In a separate study of 19 patients with NPC receiving definitive radiation with weekly CT scans, significant dosimetric variations in the target, parotids, spinal cord, and brainstem were noted mostly at fractions 5 and 15, with the authors recommending A-ART re-plans at these time points [24].

Given the wide range of variability in anatomic changes of the target structures and OARs between patients throughout radiation (as discussed in the previous section), we advocate that 1 A-ART regimen is not likely applicable to all patients. Some studies have attempted to identify baseline and dosimetric factors influencing the likelihood of a patient needing A-ART during their treatment course, with the most common factors identified being: higher initial mean parotid gland dose [36, 38, 51], larger clinical target volumes (CTV) and bulkier disease [15, 38, 52], initial weight [52], and a faster rate of weight loss [36]. Most of these predictive variables have not been validated. However, one study assessed initial mean parotid dose > 22.2Gy as a cutoff value in a validation cohort of 43 patients, but the positive predictive value was only 19% with a sensitivity of 80% [51], suggesting most patients meeting this criteria still would not benefit from A-ART, resulting in significant clinic inefficiencies.

Recent advances have looked at individualizing indications for A-ART by recalculating the cumulative dose of the target and OAR every day or every week to identify actionable changes in dosimetry which may necessitate re-planning [53, 54]. In an initial pilot study of A-ART at MDACC, Schwartz and colleagues [55] prospectively evaluated 22 patients with oropharyngeal SCC receiving definitive radiation with weekly CT dose recalculations to prompt A-ART re-planning if target coverage was poor or if OAR sparing was inadequate. All 22 patients had at least 1 adaptive re-plan and 8 had 2 re-plans with this approach. On dosimetric analysis, the ipsilateral parotid dose was reduced by 1.3 Gy (*p* = 0.002) in those receiving 1 re-plan and 4.1 Gy in those receiving 2 re-plans [21]. In a separate prospective study also utilizing weekly CT scans in patients receiving definitive RT for HNSCC [56], patients were selected for A-ART if their re-calculated plan on their weekly CT scan yielded a PTV70 or PTV60 receiving V95 < 95% or spinal cord receiving max dose > 45 Gy. This method resulted in 8 out of 10 patients being re-planned with A-ART, with 41% of the re-plans triggered in the first 2 weeks. While these early studies have predominantly used weekly CT scans, there has been recent effort to improve efficiency by utilizing CBCTs used in the daily delivery of radiation to calculate the cumulative dose received [53, 57–59] allowing the prompt identification of patients likely to benefit from A-ART. As technology and artificial intelligence advances, we anticipate that the identification of patients and the implementation of A-ART will be significantly smoother and likely automated. Table 3 describes currently accruing and upcoming trials in A-ART.

**Table 3:** Currently accruing or upcoming clinical trials in anatomy-adapted adaptive radiotherapy (A-ART)

Clinical Trial	Primary Investigator (Location)	Description	Eligible	Target Accrual (Actual or Current Accrual)	Status
Evaluation of the Automatic Deformable Recontouring on the Daily MVCT for Head and Neck Cancer Adaptive Radiotherapy (GIRAFE) [45, 60]	Laprie Anne (Institut Universitaire Du Cancer Toulouse, Oncopole, France)	Prospective phase II trial evaluating the accuracy of deformable image registration on daily MV-CTs. Deformable image registration will be compared to manual recontouring on weeks 3, 4, 5, and 6. Primary Outcome: Dice similarity coefficient Implication: if deformable image registration is reliable, may help streamline A-ART and assist with identification of those who would benefit	T3–4 and/or node > 2 cm HNSCC receiving definitive RT	48	Not yet recruiting (as of July 25, 2019)
A Prospective Non-Inferiority Trial of the Use of Adaptive Radiotherapy for Head and Neck Cancer Undergoing Radiation Therapy [45]	Jillian Tsai, MD (Memorial Sloan Kettering Cancer Center)	Prospective trial comparing LRFS in those receiving ART to historical controls with the intent of assessing non-inferiority Primary Outcome: LRFS at 2 years	HNSCC receiving definitive RT	65 [61]	Active, not recruiting (as of May 27, 2019)
MRI-guided Adaptive RadioTHerapy for reducing xerostomiA in Head and Neck Cancer (MARTHA-trial) [50]	Panagiotis Balermpas, MD (University Hospital Zurich)	Prospective trial of MRI-guided IGRT with daily MRI imaging and weekly plan adaptation, with the objective of evaluating xerostomia by LENT-SOMA and salivary flow measurements at baseline, 6, 12, and 24 months Primary Outcome: 12 month grade 2 or worse xerostomia	Stages II-IVb HNSCC receiving definitive RT	44	Not yet recruiting

### Response-adapted adaptive radiotherapy (R-ART)

In contrast to A-ART, in which the subsequent radiation re-plan essentially recapitulates the original radiation targets and doses adapted to the new anatomy, response-adapted ART is the process of changing the radiation targets and/or doses based on response to treatment. Whether the “response” is identified by CT, PET-CT, or MRI, the intent of R-ART is to either escalate the radiotherapy dose to persistent disease or reduce the dose to responding disease, leading to improved tumor control and/or reduced normal tissue toxicity.

Given that in-field recurrences are still a common pattern-of-failure in HNSCC [62–64], further treatment intensification is still needed in some patient populations, with radiation dose escalation serving as one possible paradigm. Response-adapted ART is an attractive avenue for dose escalation, since persistent or refractory disease during treatment may be directly targeted, effectively reducing the volume of disease being boosted. For example, PET-guided ART is under active investigation, with persistent radiotracer uptake early in treatment thought to represent radioresistance. Both standard tracers such as [18F]Fluoro-2-deoxy-2-D-glucose (FDG) [65, 66] and more novel indicators of hypoxia such as [18F]Fluoroazomycin-arabinoside (FAZA) [61, 67] are being studied.

Oncologic outcomes with PET-based R-ART is limited, but preliminary reports suggest that such a paradigm is feasible [68, 69]. In an initial phase I feasibility trial at Ghent University Hospital [68], the radiotherapy dose was escalated to over 80 Gy to areas of persistent avidity on a PET-CT scan performed during week 2. No acute dose-limiting toxicity was encountered. Although randomized evidence is not yet available, a recent case-matched control study [70] compared 72 patients treated on this study or similar subsequent trials receiving 70.2–85.5Gy to those receiving standard IMRT did not find a statistically significant difference in 5 year local control (82.3% vs 73.6%, *p* = 0.36). However, this retrospective analysis did note increased chronic toxicity at higher doses, with late grade > 3 dysphagia being 50% (vs 28%, *p* = 0.004) and with grade 4 mucosal ulcers occurring at the site of dose escalation in 13% (9/72) of patients [70]. The incidence of these late grade 4 mucosal ulcers was correlated with both higher hotspots in the plan (84 Gy was an identified threshold) as well as continued smoking or alcohol use following therapy [71].

It is still an open question whether dose-escalation is a reasonable approach to improve locoregional control in HNSCC, especially in this new era of immunotherapy. Although increased toxicity with dose escalation is anticipated, whether the potential for improved locoregional control counterbalances potentially serious mucosal complications remains to be seen. Fortunately, several randomized phase II trials are currently attempting to answer this question. The C-ART-2 study is a randomized phase II trial at the University Hospital of Ghent comparing its institutional R-ART dose-escalation technique (R-ART based on interim PET-CT during treatment) with standard chemoradiotherapy, with the primary endpoint being locoregional control [72]. ARTFORCE is a multi-institutional randomized phase II trial assessing if PET-guided dose-escalation to 84Gy/35Fx improves locoregional control in comparison to standard RT 70Gy/35Fx. This study uses PET to develop the initial dose-escalation volume but the adaptation is actually strictly based on CT-only changes at week 2 [73, 74].

In contrast to dose-escalation approaches to R-ART, a separate treatment philosophy is to use interim diagnostic imaging to guide dose de-intensification in responders, with the goal to improve the acute and chronic toxicity profile of HNSCC RT. Preliminary work on correlating interim PET-CT tumor response to local-regional failure free survival (LRFS) has demonstrated that, in general, patients who have a more pronounced metabolic response by mid-treatment scan appear to have better long-term locoregional control [75–77]. These non-intervention studies have generated the exciting concept that interim PET-CT can select robust responders for dose de-intensification strategies, but no prospective data are yet available to prove the viability of this paradigm. An upcoming phase II feasibility study, entitled PEARL, will be assessing if dose de-intensification of surrounding normal tissues can safely be executed with the use of an intra-treatment PET/CT at 2 weeks to guide reduction in target volumes as the tumor responds [78].

A separate approach is to harness the superior soft tissue definition of MRI imaging to continuously adapt and shrink the high-dose treatment volume to MRI-visible disease [47, 48]. Initial pilot trials utilizing intra-treatment MRI imaging in patients with OPC receiving definitive CRT have demonstrated high CR rates during treatment, with one reporting 51.7% CR of the primary by week 3–4 [47], a second reporting 90 and 100% volume reduction by weeks 4 and 6 [48], and a third reporting 70% GTV reduction by week 6 [49]. These rates of tumor shrinkage appear higher than what has been historically reported in separate studies using CT-based intra-treatment scans (see Table 1). This has led to interest of whether MRI-guided R-ART may help guide further shrinkage of high dose target volumes. However, some concern has been raised over whether limiting the target volumes to only the shrinking MRI-visible disease may hurt local control, as it has been hypothesized that at least some of the tumor may be dissolving instead of shrinking, leaving behind microscopic disease in areas previously occupied by the tumor. In a small study of 8 patients with locally advanced OPC receiving definitive CRT, fiducials were placed around the outer edge of the primary tumors and patients had repeat MRIs done during weeks 3 and 6 of CRT. They found that the GTV as detected on MRI reduced in size more than the displacement of the fiducials (absolute difference of 0.1 and 0.3 cm at weeks 3 and 6, respectively) supporting the hypothesis that some of the tumor may be dissolving [49]; this finding implies that the area previously occupied by the tumor on baseline scans may still require low dose radiation sufficient to eradicate microscopic disease. The MR-ADAPTOR trial is a currently accruing phase II study that is assessing if weekly MRI imaging can be safely used to guide adaptation of the high risk target volumes whilst maintaining coverage of the areas previously occupied by disease with at least 50.16 Gy, with the primary endpoint being determination if 6 month LRC is similar to standard non-adapted IMRT [37, 79]. Table 4 details currently accruing and upcoming trials regarding R-ART.

**Table 4:** Currently accruing or upcoming clinical trials in response-adaptive adaptive radiotherapy (R-ART)

Clinical Trial	Primary Investigator (Location)	Description	Eligible	Target Accrual (Actual or Current Accrual)	Status
PEARL PET-based Adaptive Radiotherapy Clinical Trial (PEARL) [78]	Mererid Evans (Velindre Cancer Centre, Wales, United Kingdom)	Prospective phase II feasibility study of biological dose adaptation using PET/CT at baseline and at 2 weeks Primary Outcome: PFS at 2 years	P16-positive oropharyngeal SCC T1–3 N0–1 M0 being treated with definitive CRT and non-smoker for > 2 years	50	Not yet recruiting (as of May 2, 2019)
Comparison of Adaptive Dose Painting by Numbers with Standard Radiotherapy for Head and Neck Cancer **(**C-ART-2) [72]	Wilfried de Neve, MD PhD (University Hospital, Ghent, Belgium)	Randomized phase II trial randomizing participants to adaptive dose-painting-by-numbers or to standard radiation Primary Outcome: LC at 1 year	SCC of the oral cavity, oropharynx, hypopharynx, or larynx which is T1–4 (or T3–4N0 or T1–4N1–3 if glottic) with decision for definitive RT or CRT	100 (95)	Active, not recruiting (as of May 21, 2018)
Adaptive, Image-guided, Intensity-modulated Radiotherapy for Head and Neck Cancer in the Reduced Volumes of Elective Neck: a Multicenter, Two-arm, Randomized Phase II Trial [80]	Wilfried de Neve, MD PhD (University Hospital, Ghent, B3elgium)	Randomized phase II trials randomizing participants to standard IMRT or to adaptive radiotherapy (with 2 re-scans with either CT, PET/CT, or MRI during treatment) with the objective to reduce elective neck volumes based on tumor response Primary Outcome: Reduction of acute and late dysphagia	T1–4 N0–3 HNSCC receiving definitive RT	100 (100)	Completed, not yet published (as of January 27, 2016)
Adaptive and innovative Radiation Treatment FOR improving Cancer treatment outcome (ARTFORCE) [73, 74]	Olga Hamming-Vrieze, MD (The Netherlands Cancer Institute)	Randomized phase II trial randomizing participants in a factorial 2 by 2 design to cisplatin or cetuximab and standard RT 70Gy/35Fx or adaptive radiotherapy 70-84Gy/35Fx boosting the 50% SUV max inside the GTV, with re-scans at week 2 Primary Outcomes: 2 year grade 3+ toxicity, 2 year LRFS	AJCC 7 Stage III/IV SCC of the oropharynx, oral cavity, or hypopharynx	268	Recruiting (as of September 28, 2017)
Magnetic Resonance-based Response Assessment and Dose Adaptation in Human Papilloma Virus Positive Tumors of the Oropharynx treated with Radiotherapy (MR-ADAPTOR) [37, 79]	Clifton Fuller, MD PhD (MD Anderson Cancer Center)	Phase II trial using weekly MRI imaging to assess treatment response and guide dose de-intensification by reducing the 69.96 Gy PTV volume as the tumor shrinks. Note the elective volumes will not change during R-ART and will receive a minimum of 50.16 Gy. Stage 2 will randomize participants to standard IMRT or MRI-guided RT. Primary Outcome: LRC at 6 months	P16-positive T1–2 N0-2B (AJCC 7), lymph node < 3 cm, pack years < 10, receiving definitive RT	Stage 1: 15 Stage 2: 60 Total: 75	Recruiting (as of July 26, 2019)

## Conclusions

Although significant advances in radiation delivery and image guidance have led to clear improvements in quality-of-life following head and neck radiotherapy, these methods do not account for volumetric and spatial changes that occur throughout treatment, sometimes as early as week 2. Anatomy-adapted adaptive radiotherapy (A-ART) offers a way to counteract these changes, achieving maintenance of target coverage and avoiding OAR overdosage, by re-simulating and re-planning patients either in response to a clinical or dosimetric signal or at regularly timed intervals. Currently, there is no consensus on the most appropriate way to incorporate A-ART into clinical practice. Noting the wide heterogeneity in volume and spatial changes of targets and OARs across patients, A-ART may be futile for those with minimal anatomic change, while it could be instrumental in dosimetric optimization in those with more pronounced changes. However, randomized evidence is not yet available to confirm a clinical benefit. Given the time burden required to re-plan patients and the low yield of A-ART for a subgroup of patients without much anatomic change, the identification of individuals who would most benefit is an area of active research. Perhaps, most promising is the development of automated methods for calculating cumulative dose received by the targets and OARs to identify candidates for A-ART. Soon even *clinical re-planning* will be feasible based on each CBCT [81], so that A-ART can be entirely automated. In fact, if *daily* adaptive re-planning becomes more automated and streamlined, planning target volume (PTV) expansions currently used for setup uncertainty could be significantly reduced, minimizing normal tissue doses from day one.

Response-adapted adaptive radiotherapy (R-ART) has been the subject of more recent prospective investigations and holds the promise of using novel technologies to improve tumor control and/or the acute and late tolerance of radiotherapy. Several trials utilizing R-ART should mature over the next several years and may help discern whether such an approach is worth further pursuit. In principle, response-adapted ART may further improve the therapeutic ratio in a disease site whose normal tissue structures are intrinsically entangled with the targets for irradiation.

## Data Availability

Not applicable.

## Abbreviations

A-ART: Anatomy-adapted Adaptive Radiotherapy
ART: Adaptive Radiotherapy
CBCT: Cone-Beam CT
CR: Complete Response
CT: Computed Tomography
CTV: Clinical Target Volume
FAZA: [18F]Fluoroazomycin-arabinoside
FDG: [18F]Fluoro-2-deoxy-2-D-glucose
Fx: Fraction
GTV: Gross Tumor Volume
HNSCC: Head and Neck Squamous Cell Carcinoma
Hypoph: Hyopharyngeal Cancer
IMRT: Intensity-Modulated Radiation
Laryng: Laryngeal Cancer
MRI: Magnetic Resonance Imaging
NPC: Nasopharyngeal Cancer
NPC: Nasopharyngeal Carcinoma
NS: Head and Neck Squamous Cell Carcinoma, Site Not Specified
OAR: Organ at Risk
OC: Oral Cavity Cancer
OPC: Oropharyngeal Cancer
PTV: Planned Target Volume R-ART Response-adapted Adaptive Radiotherapy
SN: Sinonasal Cancer
UP: Head and Neck Squamous Cell Carcinoma of Unknown Primary
